# Pyrrhotite Facilitates Growth and Cr Accumulation in *Leersia hexandra* Swartz for Effective Cr(VI) Removal in Constructed Wetlands

**DOI:** 10.3390/toxics14010107

**Published:** 2026-01-22

**Authors:** Xinyue Zhang, Xuehong Zhang, Yue Lin, Jiang Lv, Minmin Jiang, Sijia Cheng, Jun Yan

**Affiliations:** 1 College of Environmental Science and Engineering, Guilin University of Technology, Guilin 541004, China; 2 Science and Technology Innovation Center, Hubei Institute of Urban Geological Engineering, Wuhan 430050, China; 3 College of Life and Environmental Science, Guilin University of Electronic Technology, Guilin 541004, China; 4 School of Accounting and Auditing, Guangxi University of Finance and Economics, Nanning 530003, China; 5 Guangxi Key Laboratory of Environmental Pollution Control Theory and Technology, Guilin University of Technology, Guilin 541004, China

**Keywords:** hyperaccumulator, constructed wetland, iron–sulfur mineral, heavy metal removal, standing stock

## Abstract

Hexavalent chromium (Cr(VI)) is a hazardous pollutant frequently found in industrial wastewater. Constructed wetlands (CWs) provide an alternative for Cr(VI) removal, but their effective removal is essentially governed by the extent of Cr accumulation in plants. This study evaluated the effects of pyrrhotite addition on a Cr-hyperaccumulator *Leersia hexandra* Swartz (*L. hexandra*) in CW microcosms with different substrates (pyrrhotite and gravel) and influent Cr(VI) concentrations (2 and 10 mg·L^−1^). All microcosms achieved substantial Cr(VI) removal, while pyrrhotite significantly facilitated the removal of NO_3_^−^-N, COD, and TP. Pyrrhotite alleviated Cr-induced oxidative stress and thus promoted photosynthesis in *L. hexandra*, reflected by 27.32–39.09% lower malondialdehyde levels, 1.67–8.37% higher total chlorophyll contents, and 17.36–39.61% higher net photosynthetic rates. Consequently, maximum aboveground Cr standing stock reached 164.50 mg·m^−2^ in the P10 group, where *L. hexandra* contributed 6.63% to the total Cr removal. Microbial analysis showed reduced Cr-stress responses in pyrrhotite groups. Structural equation modeling indicated that pyrrhotite and its dissolution products promote Cr standing stock of *L. hexandra* through establishing in/ex planta defensive mechanisms. These findings provide new perspectives on phytoremediation coupled with CWs for the treatment of Cr(VI)-containing wastewater.

## 1. Introduction

Over the past few decades, the rapid expansion of industrial activities has led to serious environmental challenges. Among various pollutants, hexavalent chromium (Cr(VI)) is a particularly hazardous substance, commonly found in wastewater generated from leather tanning, electroplating, and dyeing industries [1]. Inadequately treated Cr(VI) discharges pose significant ecological risks, as Cr(VI) is highly toxic to cellular functions and known to induce mutagenesis, carcinogenesis, and teratogenesis upon exposure [2]. Therefore, the effective removal of Cr(VI) from wastewater is an urgent environmental priority.

Constructed wetlands (CWs) are a wastewater treatment technology that have been globally used for the treatment of municipal, industrial, and agricultural wastewaters [3]. Building on this, the CWs have also proven effective Cr(VI) removal, offering a cost-effective alternative to physicochemical methods, such as chemical precipitation, ion exchange, and membrane filtration [1,2,4]. In CWs, Cr(VI) can be adsorbed onto the substrate and/or reduced to Cr(III) through physicochemical and biological processes, leading to its immobilization in the wetland bed and rapid decline from the water phase [5]. However, wetland plants can be more vital for maintaining the long-term dynamic chromium pool through bioaccumulation and periodic harvesting [6]. Otherwise, excessive Cr accumulation in the wetland bed can cause chronic toxicity, potentially leading to system failure over time [5]. However, according to previous studies [7,8,9,10], common wetland plants account for only 0.5–2.0% of the total Cr removal in CWs. Currently, enhancing Cr accumulation in plants is crucial to achieve ultimately effective Cr(VI) removal in CWs.

To enhance Cr uptake by plants in CWs, the concept of phytoremediation, which refers to the use of plants for remediating soil and water environments contaminated by hazardous substances such as heavy metals [11], can be adopted. Unlike conventional plants that only require trace amounts of heavy metals for growth, candidate plants employed in phytoremediation have evolved the capability to tolerate high concentrations of specific heavy metals in their tissues via multiple physiological and molecular mechanisms [11]. Among them, *Leersia hexandra* Swartz (*L. hexandra*) is a Cr-hyperaccumulating plant species widely distributed in natural wetlands, characterized by strong adaptability and rapid growth [12,13,14]. *L. hexandra* can both passively absorb Cr(VI) via sulfate or phosphate transporters and actively uptake Cr(III) through specific iron transporter proteins [12]. However, the uptake and accumulation of Cr in *L. hexandra* occur at the cost of toxic symptoms to some extent [15]. Although it has been previously demonstrated that planting *L. hexandra* in constructed wetlands enhances Cr(VI) removal [16], effective strategies to alleviate Cr stress and boost the growth and Cr accumulation of *L. hexandra* remain necessary prior to its practical implementation.

Pyrrhotite is a widely distributed and inexpensive mineral with strong electron-donating properties, which has recently attracted considerable research attention, particularly for its potential to enhance treatment performance in CWs. For example, Shen et al. [17] demonstrated that pyrrhotite could accelerate microbial denitrification in CW microcosms, while Liang et al. [18] reported that this mineral simultaneously enhanced nitrate and phosphorus removal in a pilot-scale CW. Recent studies further indicated its promising applicability in Cr(VI)-loaded CWs [1,2,4]. However, few studies have reported the regulation of wetland plants by pyrrhotite for heavy metal purposes. Though certain iron-containing filling material (e.g., engineered magnetic nanoparticles) was found to enhance plant growth through iron supplementation [19], their efficacy under heavy metal stress has not been examined. The specific role of pyrrhotite in facilitating the growth and Cr accumulation of *L. hexandra*, along with the underlying physiological mechanisms, remains poorly understood.

Therefore, this study monitored four *L. hexandra*-planted CW microcosms with different filling materials (pyrrhotite and gravel) under two influent Cr(VI) concentrations (2 and 10 mg·L^−1^). Particular attention was given to the promotional effects of pyrrhotite on the physiological traits, growth, and Cr(VI) accumulation of *L. hexandra*. Lights were also shed on the microbial composition to further elucidate the potential influence of pyrrhotite on the CW systems. The results of this study provide new perspectives on phytoremediation coupled with CWs for the treatment of Cr(VI)-containing wastewater.

## 2. Materials and Methods

### 2.1. Microcosm Setup and Operation

**Figure 1:**
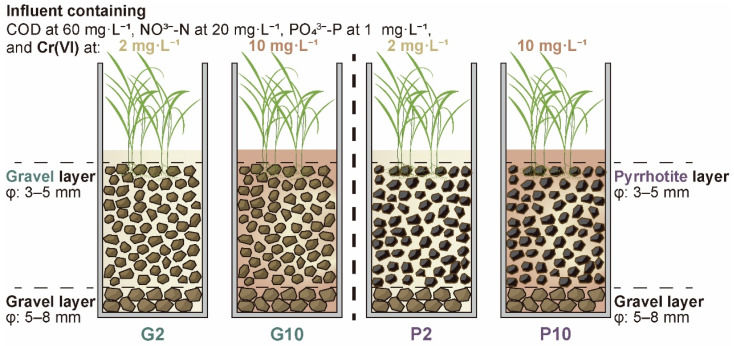
Constructed wetland microcosms filled with gravel (G2 and G10, with influent Cr(VI) concentrations of 2 and 10 mg·L^−1^, respectively) or pyrrhotite (P2 and P10).

Four groups of CW microcosms (20 cm in diameter and 50 cm in height, made of PVC, Figure 1) were established outdoors under rain-sheltered conditions with an average ambient temperature of 20 °C. For each microcosm, a 5 cm thick layer of coarse gravel (particle size: 5–8 mm) was used as the supporting material at the bottom. Two groups were then filled with pyrrhotite (particle size: 3–5 mm, Fe ~37%, S ~22%) in the reaction layer (25 cm in height) and were thus named P-groups. The other two groups were filled with gravel (particle size: 3–5 mm, SiO_2_ > 95%) in the reaction layer and were named G-groups. All filling materials were purchased from a local mining supplier and rinsed with tap water prior to use. All microcosms were planted with *L. hexandra* of uniform growth status at a density of 191 shoots·m^−2^. The microcosms were inoculated with mixed aerobic-anaerobic activated sludge (1:1 ratio) collected from Qilidian WWTP, Guilin, China. Thereafter, synthetic wastewater containing 60 mg·L^−1^ COD (sucrose), 20 mg·L^−1^ NO_3_^−^-N (potassium nitrate), and 1 mg·L^−1^ phosphorus (potassium dihydrogen phosphate) was fed in a fed-batch mode (HRT: 3 d). Additionally, different levels of influent Cr(VI) concentration (2 and 10 mg·L^−1^) were crossed with the filling material to create four specific groups (G2, G10, P2, and P10). Before the formal sampling campaign, the microcosms were pre-operated for three months to promote stable conditions.

### 2.2. Water Sampling and Analyses

The sampling campaign for influent and effluent was launched for 10 batches after the microcosms achieved stable status. A portion of the water samples was analyzed for physicochemical properties (pH, DO, ORP, and conductivity) using portable instruments (Qiwei, China). Another portion was filtered through 0.45 μm membranes and analyzed for Cr(VI), NO_3_^−^-N, COD, Fe(II), Fe(III), S^2−^, and total phosphorus (TP) using spectrophotometric methods (DR-6000, HACH, Loveland, CO, USA) according to Tu et al. [2]. Based on the results of the pre-experiment, the concentrations of total Cr and total nitrogen were essentially the same as those of Cr(VI) and NO_3_^−^-N, respectively, and thus are not reported here. SO_4_^2−^ was determined by ion chromatography (ICS-2100, Dionex, Sunnyvale, CA, USA) following Lin et al. [1].

### 2.3. Plant Sampling and Analyses

Intact plant tissues (*n* = 3) were harvested at the end of the batch experiment. They were then repeatedly rinsed with ultrapure water and EDTA under ultrasonic conditions to remove adhered heavy metals. The cleaned samples were dried at 60 °C for 72 h until constant weight was achieved. Root, stem, and leaf biomasses were separated and weighed. Subsequently, 0.2 g of each dried tissue was placed into a conical flask containing 10 mL of nitric acid and digested on a heating block at 180 °C for 2 days. The digest was then diluted to a final volume of 5 mL and analyzed for Cr, Fe, S, and P using inductively coupled plasma-optical emission spectroscopy (Optima 7000DV, PerkinElmer, Waltham, MA, USA).

The antioxidant enzyme activities, namely superoxide dismutase (SOD), catalase (CAT), and peroxidase (POD), in the leaves of *L. hexandra* (*n* = 3) were determined using commercial assay kits (BC0170, BC0200, and BC0090, Solarbio, Beijing, China) according to the manufacturer’s instructions. Malondialdehyde (MDA) content was determined by the thiobarbituric acid (TBA) colorimetric method (*n* = 3). Specifically, 0.1 g of fresh *L. hexandra* leaves from each wetland system was homogenized with 5% trichloroacetic acid (TCA) using grinding beads and then centrifuged at 4000 rpm for 10 min. Then, 2 mL of the supernatant was mixed with 2 mL of 0.6% TBA and heated in a boiling water bath for 15 min. After cooling, absorbance was measured at 480, 532, and 660 nm to calculate MDA concentration.

Chlorophyll content was determined using the ethanol extraction method (*n* = 3). Specifically, 0.1 g of fresh leaf tissue was cut into small pieces and extracted in 20 mL of a 1:1 (*v*/*v*) acetone–anhydrous ethanol mixture in the dark for 24 h. The absorbance of the resulting extract was measured at 665, 649, and 480 nm using a spectrophotometer (DR-6000, HACH, Loveland, CO, USA). The photosynthetic parameters, namely net photosynthetic rate (P_n_), stomatal conductance (G_s_), transpiration rate (T_r_), and intercellular CO_2_ concentration (C_i_), were measured in situ using a portable photosynthesis system (Yaxin-1105, Yaxin, China).

### 2.4. Microbial Sampling and Analyses

Filling materials were collected from the reaction layers of different microcosms at the end of the batch experiment. Then, biofilms (*n* = 2) were detached from the filling materials by shaking in PBS buffer. Total DNA was extracted using the E.Z.N.A.^®^ Soil DNA Kit (Omega Bio-tek, Norcross, GA, USA) according to the manufacturer’s instructions. The primers 515F (5′-GTGYCAGCMGCCGCGGTAA-3′) and 806R (5′-GGACTACNVGGGTWTCTAAT-3′) were used to amplify the bacterial 16S rRNA gene fragments (V3–V4 region). High-throughput sequencing was performed by Majorbio Bio-Pharm Technology Co., Ltd. (Shanghai, China). Details of DNA extraction, Illumina MiSeq sequencing, and amplicon sequence processing were provided in Text S1.

### 2.5. Statistical Analyses

All results were presented as the mean ± standard deviation, if applicable. Analyses of variation (ANOVA) were performed on Graphpad Prism. A threshold of *p* < 0.05 was used to determine statistical significance. The partial least squares structural equation modeling (PLS-SEM) was conducted on the basis of water and plant parameters using SmartPLS 3 software according to Chen et al. [20], while the calculation of goodness of fit (GoF) for the modeling results can be found in Text S2.

## 3. Results and Discussion

### 3.1. Overall Treatment Performance

**Figure 2:**
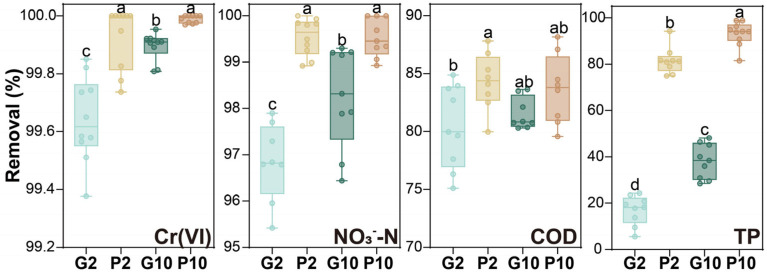
Removal of Cr(VI), NO_3_^−^-N, COD, and TP in the CW microcosms during batch experiment (*n* = 10). Different lowercase letters (a, b, c, d) indicate significant differences among groups at *p* < 0.05.

The average removal of Cr(VI), NO_3_^−^-N, COD, and TP (*n* = 10) is shown in Figure 2. Under the 2 mg·L^−1^ Cr(VI) condition, the Cr(VI) removal in the pyrrhotite and gravel groups was 99.93% ± 0.11% and 99.64% ± 0.15%, respectively. Whereas, under the 10 mg·L^−1^ Cr(VI) condition, the Cr(VI) removal in the pyrrhotite and gravel groups was 99.99% ± 0.01% and 99.89% ± 0.05%, respectively. All microcosms exhibited substantial Cr(VI) removal without significant differences, suggesting the Cr(VI) removal was not a limiting factor of their treatment performance. This observation was consistent with previous studies where efficient Cr(VI) removal was documented in different CWs [1,2,4,21], validating the general potential of CWs for Cr(VI)-contaminated wastewater treatment. Notably, the removal mechanisms of Cr(VI) removal could differ between the pyrrhotite and gravel groups. While Cr(VI) tended to be adsorbed onto filling materials and associated with organic matter in the gravel groups, pyrrhotite might further facilitate the Cr(VI) precipitation with iron oxides due to the iron release [6].

The results further indicated slight facilitation of NO_3_^−^-N removal by pyrrhotite, with significant differences between P10 (99.31% ± 0.79%) and G10 (96.68% ± 5.06%), as well as between P2 (99.54% ± 0.40%) and G2 (96.57% ± 2.06%). The role of pyrrhotite in facilitating nitrogen removal has been reported by many researchers previously. For example, Bu et al. [22] observed that within 24 h, a CW filled with pyrrhotite-modified materials reduced NO_3_^−^-N concentrations from 24 mg·L^−1^ in the influent to 5.20 mg·L^−1^ in the effluent, which was lower than that in the non-pyrrhotite control (8.62 mg·L^−1^). Liang et al. [18] found that a pyrrhotite-CW achieved 18.01% higher NO_3_^−^-N removal and 9.21% higher TN removal than the lime control, primarily due to the pyrrhotite autotrophic denitrification. Nonetheless, it was not demonstrated until a recent study that the wetland plant could contribute up to 55.5% of the pyrrhotite-facilitated TN removal in constructed wetlands [17]. Our data was also indicative of the possible role of the *L. hexandra* in utilizing pyrrhotite for nitrogen removal, with its contribution likely hindered by elevated Cr(VI) exposure.

The COD removal in P2 (81.04% ± 7.34%) and P10 (83.57% ± 4.18%) was higher than that in G2 (79.31% ± 4.90%) and G10 (80.65% ± 2.34%), although the differences were not statistically significant. As an electron-donating filling material, pyrrhotite does not necessarily promote COD removal [23]. However, iron–sulfur minerals could favor the growth of iron-respiring and sulfur-metabolizing microorganisms, which could be active in organic degradation [1]. The TP removal was 78.96% ± 9.83%, 93.19% ± 5.17%, 10.73% ± 20.69%, and 29.72% ± 27.57% in P2, P10, G2, and G10, respectively. Numerous studies have indicated that pyrrhotite and its dissolved iron products can readily bind with PO_4_^3−^ [1,22,23]. Moreover, a higher concentration of Cr(VI) in the influent did not alter the difference between the pyrrhotite and gravel groups, but it did increase TP removal. This suggested that TP removal in the CW microcosms was mainly governed by physicochemical processes and potentially related to Cr-PO_4_^3−^ precipitation.

Other water parameters were shown in Figure S1. Generally, lower concentrations of soluble sulfur species (S^2−^ and SO_4_^2−^) were detected in the effluent of the pyrrhotite groups than in the gravel groups, probably due to iron–sulfur mineralization, as indicated by the slightly elevated iron levels in the pyrrhotite groups (5.01 ± 2.43 mg·L^−1^) compared to nearly undetectable levels in the gravel groups.

### 3.2. Effect of Pyrrhotite on Physiology of L. hexandra

**Figure 3:**
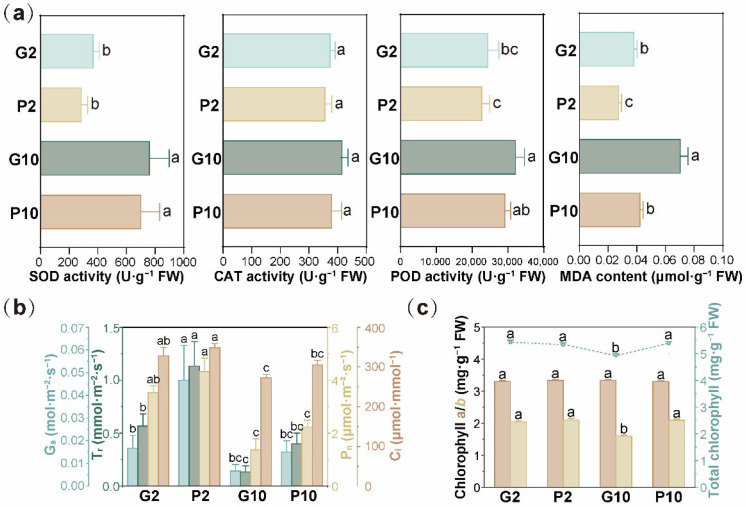
Superoxide dismutase (SOD) activity, catalase (CAT) activity, peroxidase (POD) activity, and malondialdehyde (MDA) content in leaf tissues of *L. hexandra* in the CW microcosms (**a**), the photosynthetic parameters of *L. hexandra* in the CW microcosms (**b**), and the chlorophyll contents of *L. hexandra* in the CW microcosms (**c**). All data were based on triplicated samples (*n* = 3). Different lowercase letters (a, b, c) indicate significant differences among groups at *p* < 0.05.

As shown in Figure 3a, SOD activity in *L. hexandra* rose significantly in response to higher influent Cr(VI) concentrations, with values of 374.87 ± 40.41, 291.97 ± 44.16, 767.42 ± 136.93, and 706.97 ± 132.41 U·g^−1^ FW in G2, P2, G10, and P10, respectively. No significant difference was found for CAT activity between groups, as it remained 377.93 ± 17.65, 359.79 ± 23.38, 419.14 ± 26.53, and 382.61 ± 35.42 U·g^−1^ FW in G2, P2, G10, and P10, respectively. POD activity also responded significantly to the elevated Cr(VI) exposure, with values of 24,624.30 ± 3124.90, 22,940.82 ± 2202.43, 32,408.85 ± 2465.91, and 29,385.47 ± 1587.38 U·g^−1^ FW in G2, P2, G10, and P10, respectively.

Under Cr(VI) exposure, *L. hexandra* readily accumulates Cr in its biomass. According to a previous study, Cr(VI) entering the cell is reduced through Cr(V) and Cr(IV) intermediates [15]. These intermediates trigger excessive accumulation of reactive oxygen species (ROS), causing oxidative damage to cells and ultimately impairing plant growth and development [24]. SOD is regarded as the primary defense mechanism that scavenges excessive intracellular ROS (e.g., superoxide) by catalyzing their dismutation into hydrogen peroxide (H_2_O_2_) and oxygen (O_2_). The resulting H_2_O_2_ is further decomposed into H_2_O and O_2_ by CAT and POD, thereby mitigating oxidative stress [25]. Therefore, the significantly upregulated SOD and POD activities in *L. hexandra* indicated that its antioxidant defense mechanisms were activated in response to Cr(VI) stress, which is consistent with previous studies [26,27].

The MDA content in *L. hexandra* was 0.039 ± 0.002, 0.028 ± 0.002, 0.071 ± 0.005, and 0.043 ± 0.002 μmol·g^−1^ FW in the G2, P2, G10, and P10 groups, respectively (Figure 3a). Both the increase of MDA with elevated Cr(VI) concentration and its reduction due to pyrrhotite addition were statistically significant. MDA can serve as an indicator of lipid peroxidation and a marker of oxidative damage, with its increase frequently reported when plants are exposed to Cr stress [28,29]. These results indicated that, without pyrrhotite addition, Cr(VI) concentrations of 2–10 mg·L^−1^ could exert toxic effects on *L. hexandra*; thus, its bare application in CWs for Cr(VI) removal may be ineffective. Moreover, the significantly lower MDA content, along with comparable antioxidant enzyme activities in *L. hexandra* in the pyrrhotite groups versus gravel groups, further evidenced the ex planta role of pyrrhotite in pre-defense against Cr stress.

As shown in Figure 3b, the photosynthetic parameters (P_n_, G_s_, T_r_, C_i_) were significantly suppressed under the elevated Cr(VI) condition. Particularly, P_n_ in G2, P2, G10, and P10 were 3.53 ± 0.25, 4.33 ± 0.50, 1.37 ± 0.42, and 2.23 ± 0.25 μmol·m^−2^·s^−1^, respectively. G_s_ in these four groups were 0.02 ± 0.01, 0.05 ± 0.02, 0.01 ± 0.01, and 0.02 ± 0.01 mol·m^−2^·s^−1^, respectively. T_r_ in these four groups were 0.57 ± 0.12, 1.13 ± 0.23, 0.13 ± 0.06, and 0.40 ± 0.10 mmol·m^−2^·s^−1^, respectively. C_i_ in the corresponding groups were 327.77 ± 21.43, 348.67 ± 10.44, 273.00 ± 8.49, and 305.30 ± 11.83 μmol·mmol^−1^, respectively. This indicated photosynthetic inhibition induced by Cr(VI) exposure. Nonetheless, G_s_ and T_r_ were significantly higher in P2 than in G2, suggesting that pyrrhotite alleviated Cr(VI)-induced photosynthetic stress in *L. hexandra*. The inhibiting effect of Cr on photosynthetic processes has been widely reported. For example, Pooja et al. [30] found that the P_n_, C_i_, G_s_, and T_r_ of *Brassica juncea* were significantly reduced under Cr treatment compared to the control. Kumar et al. [31] observed that Cr(VI) exposure led to a significant decline in the P_n_ and G_s_ of *Helianthus annuus*. Besides, chlorophyll contents were negatively affected by Cr(VI) exposure to some extent, as chlorophyll b was significantly lower in G10 compared with the other groups (Figure 3c). Previous studies indicated that excessive Cr accumulated in plant tissue will impair the photosynthetic systems by inhibiting Calvin cycling, disrupting electron transport, altering chloroplast structure, and causing stomatal closure [30,31]. In this regard, the pyrrhotite addition would restore the photosynthetic activity of *L. hexandra* under Cr(VI) exposure, maintaining efficient energy capture to support growth. Meanwhile, Cr accumulation by *L. hexandra* would also benefit from the enhanced photosynthetic system, as Cr uptake by this plant is considered to involve active transport that consumes energy [24].

### 3.3. Effect of Pyrrhotite on Growth and Cr Accumulation in L. hexandra

**Figure 4:**
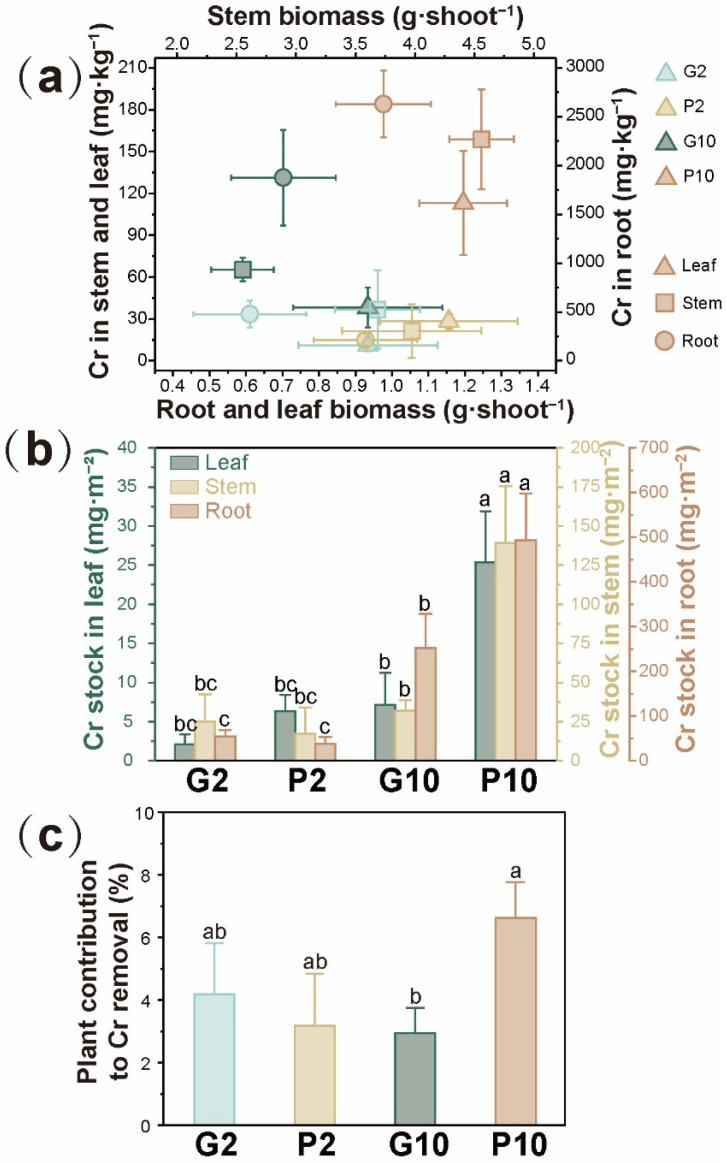
Biomass and Cr accumulation in root, stem, and leaves of *L. hexandra* (**a**), Cr stock in root, stem, and leaves of *L. hexandra* (**b**), and plant contribution to Cr removal in the CW microcosms (**c**). All data were based on triplicated samples (*n* = 3). Different lowercase letters (a, b, c) indicate significant differences among groups at *p* < 0.05.

As shown in Figure 4a, pyrrhotite significantly enhanced the biomass of *L. hexandra* in the CW microcosms. The root, stem, and leaf biomass were 0.61 ± 0.15, 3.69 ± 0.36, and 0.93 ± 0.19 g·shoot^−1^ in the G2 group and 0.93 ± 0.14, 3.97 ± 0.59, and 1.16 ± 0.19 g·shoot^−1^ in the P2 group, respectively. Moreover, the root, stem, and leaf biomass were 0.70 ± 0.14, 2.55 ± 0.26, and 0.93 ± 0.21 g·shoot^−1^ in the G10 group, respectively, suggesting that stem biomass was significantly reduced under elevated Cr(VI) exposure in the gravel groups. However, with pyrrhotite, the biomass was not negatively affected and was even slightly enhanced by elevated Cr(VI) exposure, with root, stem, and leaf biomass of 0.98 ± 0.13, 4.56 ± 0.27, and 1.20 ± 0.12 g·shoot^−1^, respectively, in the P10 group. Coincidentally, the iron stock in the stem and root of *L. hexandra* was significantly enriched, particularly under 10 mg Cr(VI)·L^−1^ condition (Figure S2). The iron contained in pyrrhotite is generally recognized as one of the essential microelements that affect plant growth, particularly in the photosynthetic system [32]. Besides, *L. hexandra* in the pyrrhotite groups was found to contain more phosphorus than in the gravel groups, suggesting enhanced nutrient levels and growth status induced by pyrrhotite (Figure S2). These findings were in line with the resilient chlorophyll b in the P10, as revealed in Figure 3c. Moreover, pyrrhotite enhanced plant tolerance to Cr by decreasing MDA content (Figure 3a), which was consistent with the findings of Tang et al. [33].

A more remarkable finding was the substantial enhancement of Cr accumulation by *L. hexandra* in the P10 compared with the G10, despite no significant difference between the P2 and G2 (Figure 4a). Cr accumulation in root, stem, and leaf tissues was 478.63 ± 135.54, 36.82 ± 27.93, and 11.20 ± 4.32 mg·kg^−1^ DW in the G2 group and 213.87 ± 63.90, 21.30 ± 19.28, and 28.27 ± 5.60 mg·kg^−1^ DW in the P2 group, respectively. As the influent Cr(VI) concentration increased to 10 mg·L^−1^, the accumulation increased to 1877.15 ± 489.36, 65.30 ± 8.44, and 38.19 ± 14.48 mg·kg^−1^ DW in the G10 group and 2629.00 ± 341.07, 158.75 ± 35.75, and 112.99 ± 37.26 mg·kg^−1^ DW in the P10 group, respectively. According to van der Ent et al. [34], plants that meet the criterion of accumulating at least 300 mg Cr·kg^−1^ DW in aboveground tissues can be classified as Cr hyperaccumulators. Although previous studies have demonstrated the substantial Cr accumulation capability of *L. hexandra* in its root, stem, and leaf tissues [24,35], only about half of the Cr hyperaccumulation criterion was reached in the present study. This was probably due to the shorter growth period (30 d) compared with the 90 to 240 d required for achieving hyperaccumulation in previous studies [36,37]. Additionally, the relatively low Cr(VI) concentration in the influent might have limited Cr accumulation in *L. hexandra*, as no significant negative effect on biomass was observed in the P10, and the biomass was even promoted under the 10 mg·L^−1^ Cr(VI) condition. Nonetheless, our results suggest that pyrrhotite holds untapped potential to enhance Cr accumulation in *L. hexandra*.

Many plants have been reported to exhibit extremely high accumulation of heavy metals, but their growth is often restricted due to toxic effects. Therefore, Jan and Tereza [6] argued that standing stock (i.e., the total storage of a substance in a particular compartment of CWs) provides a more rational metric for evaluating the efficacy of heavy metal accumulation by wetland plants. In this context, Figure 4b offered a more straightforward perspective illustrating the role of pyrrhotite in promoting Cr accumulation by *L. hexandra*. As pyrrhotite simultaneously promoted the biomass and Cr accumulation of *L. hexandra*, the Cr standing stocks in root, stem, and leaf reached 492.85 ± 104.66, 139.12 ± 36.33, and 25.38 ± 6.52 mg·m^−2^, respectively, in the P10, which were remarkably higher than those in the G10 (251.79 ± 76.12, 31.99 ± 6.88, and 7.16 ± 4.12 mg·m^−2^), P2 (38.41 ± 14.59, 17.15 ± 17.03, and 6.35 ± 2.04 mg·m^−2^), and G2 (54.14 ± 15.07, 24.97 ± 17.65, and 2.10 ± 1.26 mg·m^−2^). According to a previous review, the Cr standing stock in the aboveground biomass of a typical wetland plant, *Phragmites australis*, ranged from 0.1 to 24.3 mg·m^−2^ [6]. By comparison, our results suggested that the combination of pyrrhotite and *L. hexandra* could achieve a Cr standing stock up to 6.77 times higher than the reported maximum value, highlighting its great potential for effective Cr removal in CW applications. Notably, as an emergent plant with a shallow root system that can be easily harvested, *L. hexandra* also exhibited the large Cr standing stock in its belowground biomass, further contributing to its utilization in CWs [35].

Figure 4c showed the percentage of Cr sequestered in the total biomass of *L. hexandra* relative to the total Cr mass removal. *L. hexandra* contributed 3.18% ± 1.67%, 4.18% ± 1.63%, 6.63% ± 1.13%, and 2.94% ± 0.18% to Cr removal in P2, G2, P10, and G10, respectively. Worth mentioning, given the limited Cr remaining in the effluent, the higher Cr removal contribution of *L. hexandra* might indicate a proportionally reduced Cr sink in wetland sediments [38], which in turn pointed to a healthier status of the CW system. In comparison with the previously documented contribution range (0.5–2.0%) of common wetland plants to Cr removal in CWs [7,8,9,10], pyrrhotite-amended CWs have improved the Cr removal contribution of *L. hexandra* in this study. Moreover, the facilitating role of pyrrhotite was more pronounced under higher Cr(VI) exposure. However, there is considerable room remaining for promoting *L. hexandra* contribution to Cr removal. The current density (191 shoots·m^−2^) was conservative and far lower than that reported for mature CWs planted with, for example, *Schoenoplectus tabernaemontani* and *Juncus roemerianus* (up to 2000 shoots·m^−2^) [39], and increasing plant density could be a practical strategy.

### 3.4. Effect of Pyrrhotite on Microbial Community Structure

**Figure 5:**
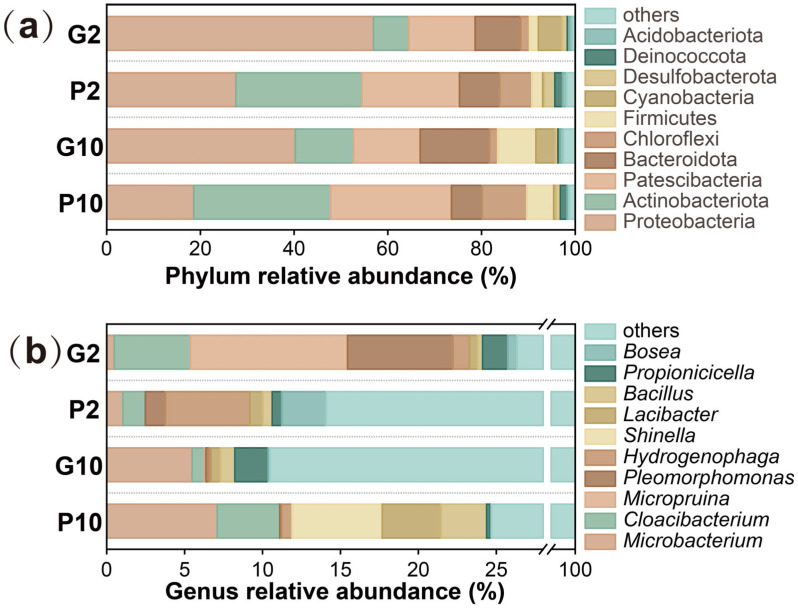
Relative abundances on phylum (**a**) and genus (**b**) level of microbial samples.

According to the 16S rRNA high-throughput sequencing results, *Proteobacteria*, *Actinobacteriota*, *Patescibacteria*, *Bacteroidota*, and *Chloroflexi* were the dominant phyla in the CW microcosms (Figure 5a). *Proteobacteria* have been recognized as an important phylum involved in the biological removal of nitrogen and organic pollutants [40]. Besides, it has also been found dominant in some Cr(VI)-contaminated soils and aquatic environments [41,42], exhibiting the capability to biotransform Cr(VI) into Cr(III) [43]. However, it was found that *Proteobacteria* were more enriched in G10 (40.24%) and G2 (56.94%) than in P10 (18.57%) and P2 (27.57%). In contrast, *Actinobacteriota*, *Patescibacteria*, and *Chloroflexi* were more abundant in the pyrrhotite groups (P10: 29.22%, 25.79%, and 9.56%; P2: 26.88%, 20.86%, and 6.86%) than in the gravel groups (G10: 12.44%, 14.24%, and 1.76%; G2: 7.53%, 14.16%, and 1.90%). These phyla are commonly associated with denitrifying environments [1]. According to Kong et al. [4], the enrichment of Cr(VI)-reducing microbes can be attributed to Cr(VI) stress in the environment. Thus, it is reasonable to infer that pyrrhotite likely immobilized Cr(VI), most probably via chemical reduction and precipitation [5], prior to the accession of Cr(VI) to the microbial community. Consequently, this alleviated the selective pressure on the microbial community to evolve dedicated defensive mechanisms against Cr(VI). Yet, these inferred mechanisms require further validation in future studies.

At the genus level, the relative abundances of *Microbacterium*, *Lacibacter*, *Bosea*, and *Bacillus* were significantly higher in gravel groups than in pyrrhotite groups. Specifically, their abundances accounted for 1.04%, 0.83%, 2.86%, and 0.61% in G2, compared with 0.49%, 0.52%, 0.60%, and 0.33% in P2, respectively, and 7.10%, 3.78%, 0.10%, and 2.92% in G10, compared with 5.48%, 0.61%, 0.10%, and 0.92% in P10, respectively. Concurrently, these microbes were more abundant under higher Cr(VI) stress (Figure 5b). These genera have been recognized for their roles in Cr(VI) reduction, Cr(VI) biosorption, or their association with Cr-contaminated environments [44,45,46,47]. By contrast, pyrrhotite favored the growth of *Micropruina* (0.03%, 10.09%, 0.00%, and 0.10% in G2, P2, G10, and P10, respectively) and *Pleomorphomonas* (1.28%, 6.76%, 0.11%, and 0.21% in G2, P2, G10, and P10, respectively), which happened to be suppressed under higher Cr(VI) stress. These genera are known for their roles in denitrification or nitrogen metabolism [48,49]. Apparently, the microbial communities in gravel groups underwent heavy selection for enhanced Cr(VI) tolerance. In contrast, their counterparts in pyrrhotite groups might have been subject to lower selective pressure. Consequently, a more diverse microbial community grew in the pyrrhotite-amended groups (Table S1). These results were consistent with those obtained at the phylum level, further demonstrating that pyrrhotite can act as a stress-buffering agent to protect microbial communities from Cr(VI) stress. Nonetheless, future studies should further elucidate the functional differences of the microbial community to validate the stress-buffering mechanism of pyrrhotite.

### 3.5. Facilitating Mechanism of Pyrrhotite in L. hexandra Application

**Figure 6:**
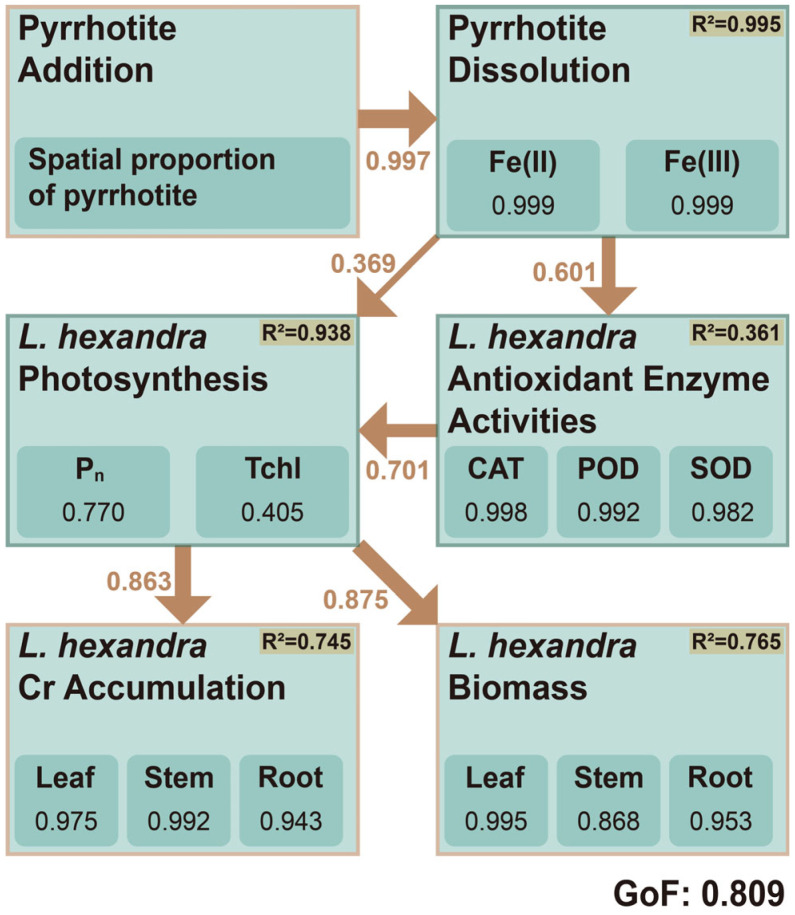
Partial least squares structural equation modeling of pyrrhotite-mediated effects on biomass and Cr accumulation in *L. hexandra*. Path coefficients are shown on arrows; R^2^ values indicate explained variance; goodness of fit (GoF) indicates the adaptation degree of this model.

As shown in Figure 6, the PLS-SEM illustrated the mechanisms through which pyrrhotite facilitated Cr accumulation and biomass in *L. hexandra*. The model yielded a GoF of 0.809 (acceptable GoF > 0.36), indicating good overall fit [50,51]. According to the result, pyrrhotite provided an excellent explanation for the increase in Fe(II) and Fe(III) contents in the microcosms (path coefficient: 0.997; R^2^: 0.995), indicating the pyrrhotite dissolution process. Nonetheless, the sulfur species could also have been released along with their iron counterpart during this process. However, only iron contents were considered representative of pyrrhotite dissolution in the model, as sulfur species are unstable and prone to reprecipitation (Figure S1), which obscures a clear understanding of pyrrhotite reactions.

Subsequently, the antioxidant enzyme activities of *L. hexandra* could be influenced by pyrrhotite dissolution (path coefficient: 0.601) with an overall R^2^ of 0.361. Several possible mechanisms were proposed for this. First, pyrrhotite and its released Fe(II) species could reduce Cr(VI) in the substrate matrices of CWs [5]. Second, the iron (oxihydr)oxides could co-precipitate with Cr(VI), particularly on root surface to form tight iron plaque that further block the direct uptake of toxic Cr(VI) species by the plants [52]. Instead, the transporters for Fe(III) complexes in *L. hexandra* might be upregulated in iron-rich pyrrhotite-amended groups to enhance the unintentional uptake of less toxic Cr(III), owing to the similar chemical properties of Cr(III) and Fe(III) [12]. This was consistent with the higher iron accumulation of *L. hexandra* in pyrrhotite groups than in gravel groups (Figure S2). Besides, the iron-rich pyrrhotite-filled environment might also upregulate the Fe(III) reductase of the plant, which was proposed to be able to catalyze Cr(VI) reduction to Cr(III) in the root [12]. These processes would have mitigated the toxic effect of Cr(VI) on *L. hexandra*. As a result, MDA accumulation in the pyrrhotite-amended groups was significantly lower than that in the gravel-amended groups.

The photosynthetic performance of *L. hexandra*, as reflected by net photosynthetic rate (P_n_) and total chlorophyll content (Tchl), benefited more indirectly via strengthened antioxidant enzyme activity (path coefficient: 0.701) than directly via pyrrhotite dissolution (path coefficient: 0.369), with an overall R^2^ of 0.938. Pyrrhotite could promote plant photosynthesis via iron supplementation, as iron activates chlorophyll-synthesizing enzymes, constitutes core components of the photosynthetic electron transport chain, and enhances the activity of Calvin cycle-limiting enzymes [53]. However, our results suggested that pyrrhotite amendment facilitated photosynthesis in *L. hexandra* primarily through alleviating oxidative damage, as evidenced by the higher path coefficient. Consequently, with facilitated photosynthesis that produced sufficient energy, *L. hexandra* exhibited higher Cr accumulation (path coefficient: 0.863; R^2^: 0.745) and biomass yield (path coefficient: 0.875; R^2^: 0.765).

Collectively, the modeling results illustrated the facilitating mechanisms of Cr standing stock of *L. hexandra*, with iron playing a substantial role in the pyrrhotite-based CWs. However, due to the instability of sulfur species, this study remained insufficient in discussion on the specific role of sulfur as another essential component of pyrrhotite. Yet, we tentatively propose two possible mechanisms based on existing literature. First, reduced sulfur (e.g., S^2−^) may facilitate Cr(VI) reduction in wetland sediment matrices [2,29]. Second, oxidized sulfur (e.g., SO_4_^2−^) might compete for Cr(VI) uptake transporters due to their similar chemical properties [54]. Both pathways would establish sulfur-based mechanisms to further improve the effectiveness of *L. hexandra* in CWs, though these remain to be verified. Admittedly, these mechanisms were based on modeling results and thus require further validation through experimental investigations in future studies.

## 4. Conclusions

This study demonstrated the feasibility of pyrrhotite addition for improving the phytoremediation performance of *L. hexandra* in CWs treating Cr(VI)-contaminated wastewater. The major findings were as follows:

(1) Both pyrrhotite-based and gravel-based CW microcosms achieved substantial Cr(VI) removal under the 2 and 10 mg Cr(VI)·L^−1^ conditions. However, the pyrrhotite groups exhibited elevated removal of NO_3_^−^-N, COD, and TP compared with the gravel groups.

(2) Pyrrhotite alleviated Cr-induced oxidative stress in *L. hexandra*, as evidenced by reduced MDA accumulation in plant tissues. SOD and POD activities were significantly upregulated under elevated Cr(VI) exposure, yet no significant differences in antioxidant enzyme activities were observed between pyrrhotite and gravel groups.

(3) Pyrrhotite promoted the photosynthetic performance of *L. hexandra*, as evidenced by significantly higher G_s_ and T_r_ in the P2 group relative to the G2 group. Total chlorophyll content remained stable in the P10 group under elevated Cr(VI) exposure, whereas it decreased significantly in the G10 group.

(4) Pyrrhotite substantially facilitated the growth and Cr(VI) accumulation of *L. hexandra*, with this facilitation being more profound under the 10 mg Cr(VI)·L^−1^ condition. Consequently, the maximum aboveground Cr standing stock reached 164.50 mg·m^−2^ in the P10 group, where *L. hexandra* contributed 6.63% to the total Cr removal.

(5) Pyrrhotite promoted more diverse microbial communities involved in nitrogen and organic matter transformation, whereas the gravel groups harbored more Cr-transforming microbes, likely due to the lack of a Cr-stress buffering effect.

(6) A PLS-SEM model revealed the potential mechanisms through which *L. hexandra* benefited from pyrrhotite addition. Based on the calculations, we tentatively propose that iron released from pyrrhotite enabled in/ex planta defensive mechanisms, thereby facilitating the growth and Cr accumulation of *L. hexandra* without inducing severe toxic effects in the Cr(VI)-loaded CW environment.

It should be noted that the release of iron and sulfur species remained stably low throughout the experiment and that the pH remained near neutral (Figure S1). Thus, no adverse effects were observed when pyrrhotite was applied as a filling material. Due to its lower cost and greater accessibility compared with existing remediation strategies (e.g., chelators and organic amendments), the use of pyrrhotite is considered a feasible approach to facilitate the application of phytoremediation in Cr(VI)-contaminated CWs.

However, several limitations remained in the current study, highlighting the need for further investigation. First, long-term monitoring was not conducted; thus, the sustainability of the combined effect of pyrrhotite and *L. hexandra* should be further verified. Second, the in/ex planta Cr speciation and Cr microdistribution in the plant were not characterized, and further investigation is therefore required to unravel the pyrrhotite-mediated detoxification and defensive mechanisms. Moreover, future studies should further investigate plant management strategies (e.g., harvesting protocols and frequency) and clarify biomass turnover regimes to optimize the application of *L. hexandra* in CWs.

Together, these findings highlighted the potential of integrating pyrrhotite with *L. hexandra* to enhance the effective removal of Cr(VI) in CWs.

## Data Availability

The data that support the findings of this study are available on request from the corresponding author.

## Supplementary Materials

The following supporting information can be downloaded at: https://www.mdpi.com/article/10.3390/toxics14010107/s1, Figure S1: Concentration of S^2−^, SO_4_^2−^, Fe(II), Fe(III), and DO, pH, ORP, and EC in the CW microcosms during batch experiment (*n* = 10); Figure S2: Biomass and phosphorus (a), iron (c), and sulfur (e) accumulation in root, stem, and leaves of *L. hexandra*. Phosphorus (b), iron (d), and sulfur (f) stock in root, stem, and leaves of *L. hexandra*; Table S1: Bacterial community diversity indices in CWs; Text S1: Details of DNA extraction, Illumina MiSeq sequencing, and amplicon sequence processing; Text S2: The calculation of goodness-of-fit (GoF) in the PLS-SEM model. References [50,55,56,57,58] are cited in the Supplementary Materials.

## References

[1] Lin Y., Lin H., Jiang M., Lv J., Zhang X., Yan J. (2025). Multiple electron transfer processes facilitate the simultaneous removal of NO_3_^−^-N and Cr(VI) in a pyrrhotite-based mixotrophic constructed wetland. J. Water Process Eng..

[2] Tu M., Lin H., Zhang X., Zhang X., Yan J. (2025). Elemental sulfur facilitates co-metabolism of Cr(VI) and nitrate by autotrophic denitrifiers in constructed wetlands. J. Hazard. Mater..

[3] Wu H., Wang R., Yan P., Wu S., Chen Z., Zhao Y., Cheng C., Hu Z., Zhuang L., Guo Z. (2023). Constructed wetlands for pollution control. Nat. Rev. Earth Environ..

[4] Kong Q., Guo W., Sun R., Qin M., Zhao Z., Du Y., Zhang H., Zhao C., Wang X., Zhang R. (2021). Enhancement of chromium removal and energy production simultaneously using iron scrap as anodic filling material with pyrite-based constructed wetland-microbial fuel cell. J. Environ. Chem. Eng..

[5] Jiang K., Zhang J., Deng Z., Barnie S., Chang J., Zou Y., Guan X., Liu F., Chen H. (2021). Natural attenuation mechanism of hexavalent chromium in a wetland: Zoning characteristics of abiotic and biotic effects. Environ. Pollut..

[6] Vymazal J., Březinová T. (2016). Accumulation of heavy metals in aboveground biomass of Phragmites australis in horizontal flow constructed wetlands for wastewater treatment: A review. Chem. Eng. J..

[7] Shweta S., Saswati C. (2020). Performance of organic substrate amended constructed wetland treating acid mine drainage (AMD) of North-Eastern India. J. Hazard. Mater..

[8] Mustapha H.I., van Bruggen J.J.A., Lens L.P.N. (2018). Fate of heavy metals in vertical subsurface flow constructed wetlands treating secondary treated petroleum refinery wastewater in Kaduna, Nigeria. Int. J. Phytoremediat..

[9] Lesage E., Rousseau D.P.L., Meers E., Tack F.M.G., De Pauw N. (2007). Accumulation of metals in a horizontal subsurface flow constructed wetland treating domestic wastewater in Flanders, Belgium. Sci. Total Environ..

[10] Fibbi D., Doumett S., Lepri L., Checchini L., Gonnelli C., Coppini E., Del Bubba M. (2012). Distribution and mass balance of hexavalent and trivalent chromium in a subsurface, horizontal flow (SF-h) constructed wetland operating as post-treatment of textile wastewater for water reuse. J. Hazard. Mater..

[11] Acharya A., Bellaloui N., Pilipovic A., Perez E., Maddox-Mandolini M., Fuente H.D.L. (2025). Current Assessment and Future Perspectives on Phytoremediation of Heavy Metals. Plants.

[12] Malaviya P., Singh A., Anderson T.A. (2020). Aquatic phytoremediation strategies for chromium removal. Rev. Environ. Sci. Biotechnol..

[13] Chen M., Jiang P., Zhang X., Sunahara G.I., Liu J., Yu G. (2024). Physiological and biochemical responses of *Leersia hexandra* Swartz to nickel stress: Insights into antioxidant defense mechanisms and metal detoxification strategies. J. Hazard. Mater..

[14] Zhang X., Liu J., Huang H., Chen J., Zhu Y., Wang D. (2007). Chromium accumulation by the hyperaccumulator plant *Leersia hexandra* Swartz. Chemosphere.

[15] Huang Q., Zhao J., Wang J., Yang L., Xu Y., Yu G., Bai S., Liu L. (2024). Enhancement of iron-loaded sludge biochar on Cr accumulation in *Leersia hexandra* swartz: Hydroponic test. J. Environ. Manag..

[16] Chen H., Gao B., Guo Y., Yu Q., Hu M., Zhang X. (2024). Adding carbon sources to the substrates enhances Cr and Ni removal and mitigates greenhouse gas emissions in constructed wetlands. Environ. Res..

[17] Shen C., Zhao Y., Liu W., Liu R., Zhang F., Shi Y., Wang J., Tang Q., Yang Y., Man Y. (2023). Plants boost pyrrhotite-driven nitrogen removal in constructed wetlands. Bioresour. Technol..

[18] Liang Y., Wei D., Hu J., Zhang J., Liu Z., Li A., Li R. (2020). Glyphosate and nutrients removal from simulated agricultural runoff in a pilot pyrrhotite constructed wetland. Water Res..

[19] Tombuloglu H., Slimani Y., Tombuloglu G., Alshammari T., Almessiere M., Korkmaz A.D., Baykal A., Samia A.C.S. (2020). Engineered magnetic nanoparticles enhance chlorophyll content and growth of barley through the induction of photosystem genes. Environ. Sci. Pollut. Res..

[20] Chen M., Liu S., Bi M., Yang X., Deng R., Chen Y. (2022). Aging behavior of microplastics affected DOM in riparian sediments: From the characteristics to bioavailability. J. Hazard. Mater..

[21] Jia L., Liu H., Kong Q., Li M., Wu S., Wu H. (2020). Interactions of high-rate nitrate reduction and heavy metal mitigation in iron-carbon-based constructed wetlands for purifying contaminated groundwater. Water Res..

[22] Bu Y., Song M., Huang G., Chen C., Li R. (2025). High-rate nitrogen and phosphorus removal in a sulfur and pyrrhotite modified foam concrete constructed wetland. Bioresour. Technol..

[23] Ge Z., Wei D., Zhang J., Hu J., Liu Z., Li R. (2019). Natural pyrite to enhance simultaneous long-term nitrogen and phosphorus removal in constructed wetland: Three years of pilot study. Water Res..

[24] Chen M., Zhang X., Jiang P., Liu J., You S., Lv Y. (2022). Advances in heavy metals detoxification, tolerance, accumulation mechanisms, and properties enhancement of *Leersia hexandra* Swartz. J. Plant Interact..

[25] Jia X., Zhang B., Han Y., Guan J., Gao H., Guo P. (2025). Role of reactive oxygen species (ROS) on biochar enhanced chromium phytoremediation in the soil-plant system: Exploration on detoxification mechanism. Environ. Int..

[26] Kour J., Bhardwaj T., Chouhan R., Singh A.D., Gandhi S.G., Bhardwaj R., Alsahli A.A., Ahmad P. (2024). Phytomelatonin maintained chromium toxicity induced oxidative burst in Brassica juncea L. through improving antioxidant system and gene expression. Environ. Pollut..

[27] Fu Y., Lin Y., Deng Z., Chen M., Yu G., Jiang P., Zhang X., Liu J., Yang X. (2024). Transcriptome and metabolome analysis reveal key genes and metabolic pathway responses in *Leersia hexandra* Swartz under Cr and Ni co-stress. J. Hazard. Mater..

[28] Huang Q., Ayyaz A., Farooq M.A., Zhang K., Chen W., Hannan F., Sun Y., Shahzad K., Ali B., Zhou W. (2024). Silicon dioxide nanoparticles enhance plant growth, photosynthetic performance, and antioxidants defence machinery through suppressing chromium uptake in *Brassica napus* L.. Environ. Pollut..

[29] Ahmad R., Ali S., Rizwan M., Dawood M., Farid M., Hussain A., Wijaya L., Alyemeni M.N., Ahmad P. (2020). Hydrogen sulfide alleviates chromium stress on cauliflower by restricting its uptake and enhancing antioxidative system. Physiol. Plant.

[30] Sharma P., Bakshi P., Chouhan R., Gandhi S.G., Kaur R., Sharma A., Bhardwaj R., Alsahli A.A., Ahmad P. (2025). Combined application of earthworms and plant growth promoting rhizobacteria improve metal uptake, photosynthetic efficiency and modulate secondary metabolites levels under chromium metal toxicity in *Brassica juncea* L.. J. Hazard. Mater..

[31] Kumar D., Dhankher O.P., Tripathi R.D., Seth C.S. (2023). Titanium dioxide nanoparticles potentially regulate the mechanism(s) for photosynthetic attributes, genotoxicity, antioxidants defense machinery, and phytochelatins synthesis in relation to hexavalent chromium toxicity in *Helianthus annuus* L.. J. Hazard. Mater..

[32] Briat J.-F., Dubos C., Gaymard F. (2015). Iron nutrition, biomass production, and plant product quality. Trends Plant Sci..

[33] Tang X., Wen J., Mu L., Gao Z., Weng J., Li X., Hu X. (2023). Regulation of arsenite toxicity in lettuce by pyrite and glutamic acid and the related mechanism. Sci. Total Environ..

[34] van der Ent A., Baker A.J.M., Reeves R.D., Joseph P.A., Henk S. (2012). Hyperaccumulators of metal and metalloid trace elements: Facts and fiction. Plant Soil.

[35] Zhang X., Lin Y., Lin H., Yan J. (2024). Constructed wetlands and hyperaccumulators for the removal of heavy metal and metalloids: A review. J. Hazard. Mater..

[36] Shi Y., Tang G., You S., Jiang P. (2023). Effect of External Aeration on Cr (VI) Reduction in the *Leersia hexandra* Swartz Constructed Wetland-Microbial Fuel Cell System. Appl. Sci..

[37] Shi Y., Liu Q., Wu G., Zhao S., Li Y., You S., Huang G. (2024). Removal and reduction mechanism of Cr (VI) in *Leersia hexandra* Swartz constructed wetland-microbial fuel cell coupling system. Ecotoxicol. Environ. Saf..

[38] Liu J., Li G., Shao W., Xu J., Wang D. (2010). Variations in Uptake and Translocation of Copper, Chromium and Nickel Among Nineteen Wetland Plant Species. Pedosphere.

[39] Kadlec R.H., Wallace S. (2008). Treatment Wetlands.

[40] Wu H., Yang T., Zhang M., Li A., Huang D., Xing Z. (2023). Effect of HRT on nitrogen removal from low carbon source wastewater enhanced by slurry and its mechanism. Chem. Eng. J..

[41] Chaudhari A., Paul D., Thamke V., Bagade A., Bapat V.A., Kodam K.M. (2022). Concurrent removal of reactive blue HERD dye and Cr(VI) by aerobic bacterial granules. J. Clean. Prod..

[42] Singh A., Varma A., Prasad R., Porwal S. (2022). Bioprospecting uncultivable microbial diversity in tannery effluent contaminated soil using shotgun sequencing and bio-reduction of chromium by indigenous chromate reductase genes. Environ. Res..

[43] Wang H., Zhang S., Wang J., Song Q., Zhang W., He Q., Song J., Ma F. (2018). Comparison of performance and microbial communities in a bioelectrochemical system for simultaneous denitrification and chromium removal: Effects of pH. Process Biochem..

[44] Zhao Y., Wang Q., Yang Z., Jia X., Cabrera J., Ji M. (2022). Bio-capture of Cr(VI) in a denitrification system: Electron competition, long-term performance, and microbial community evolution. J. Hazard. Mater..

[45] Koner S., Chen J., Hseu Z., Chang E., Chen K., Asif A., Hsu B. (2024). An inclusive study to elucidation the heavy metals-derived ecological risk nexus with antibiotic resistome functional shape of niche microbial community and their carbon substrate utilization ability in serpentine soil. J. Environ. Manag..

[46] Zhang H., Liu L., Chang Q., Wang H., Yang K. (2015). Biosorption of Cr(VI) ions from aqueous solutions by a newly isolated *Bosea* sp strain Zer-1 from soil samples of a refuse processing plant. Can. J. Microbiol..

[47] Guo S., Xiao C., Zheng Y., Li Y., Chi R. (2021). Removal and potential mechanisms of Cr(VI) contamination in phosphate mining wasteland by isolated *Bacillus megatherium* PMW-03. J. Clean. Prod..

[48] Xu Q., Lu S., Yuan T., Zhang F., Wang L., Wang P., Wen X., Cui L. (2021). Influences of dimethyl phthalate on bacterial community and enzyme activity in vertical flow constructed wetland. Water.

[49] Long Y., Yu G., Wang J., Zheng D. (2024). Cadmium removal by constructed wetlands containing different substrates: Performance, microorganisms and mechanisms. Bioresour. Technol..

[50] Wetzels M., Odekerken-Schroder G., van Oppen C. (2009). Using PLS path modeling for assessing hierarchical construct models: Guidelines and empirical illustration. MIS Q..

[51] Guo F., Luo Y., Nie M., Zheng F., Zhang G., Chen Y. (2023). A comprehensive evaluation of biochar for enhancing nitrogen removal from secondary effluent in constructed wetlands. Chem. Eng. J..

[52] Zhang X., Su C., Zhang Y., Lai S., Han S., Zhang X., Zheng J. (2023). Mineralogical characteristics of root iron plaque and its functional mechanism for regulating Cr phytoextraction of hyperaccumulator Leersia hexandra Swartz. Environ. Res..

[53] Kroh G.E., Pilon M. (2020). Regulation of Iron Homeostasis and Use in Chloroplasts. Int. J. Mol. Sci..

[54] Vecchia F.D., Nardi S., Santoro V., Pilon-Smits E., Schiavon M. (2023). *Brassica juncea* and the Se-hyperaccumulator *Stanleya pinnata* exhibit a different pattern of chromium and selenium accumulation and distribution while activating distinct oxidative stress-response signatures. Environ. Pollut..

[55] Magoc T., Salzberg S.L. (2011). FLASH: Fast length adjustment of short reads to improve genome assemblies. Bioinformatics.

[56] Chen S., Zhou Y., Chen Y., Gu J. (2018). fastp: An ultra-fast all-in-one FASTQ preprocessor. Bioinformatics.

[57] Callahan B.J., McMurdie P.J., Rosen M.J., Han A.W., Johnson A.J.A., Holmes S.P. (2016). DADA2: High-resolution sample inference from Illumina amplicon data. Nat. Methods.

[58] Bolyen E., Rideout J.R., Dillon M.R., Bokulich N., Abnet C.C., Al-Ghalith G.A., Alexander H., Alm E.J., Arumugam M., Asnicar F. (2019). Reproducible, interactive, scalable and extensible microbiome data science using QIIME 2. Nat. Biotechnol..

