# Supplementary material for: Pyrrhotite Facilitates Growth and Cr Accumulation in Leersia hexandra Swartz for Effective Cr(VI) Removal in Constructed Wetlands
Source: Toxics. 2026 Jan 22;14(1):107. doi: 10.3390/toxics14010107 (PMC12845952; doi:10.3390/toxics14010107)
Supplement: Supplementary file 1 [file toxics-14-00107-s001.zip › toxics-4075073-supplementary.pdf]

Supplementary Information for

Pyrrhotite facilitates growth and Cr accumulation in *Leersia hexandra* Swartz for effective Cr(VI) removal in constructed wetlands

Xinyue Zhang[1†], Xuehong Zhang[1†], Yue Lin[1], Jiang Lv[2], Minmin Jiang[3], Sijia Cheng[4,*], Jun Yan[1,5,*]

[1] College of Environmental Science and Engineering, Guilin University of Technology, Guilin, 541004, P.R. China

[2] Science and Technology Innovation Center of Hubei Institute of Urban Geological Engineering, Wuhan, 430050, P.R. China.

[3] College of Life and Environmental Science, Guilin University of Electronic Technology, Guilin, 541004, P.R. China

[4] School of Accounting and Auditing, Guangxi University of Finance and Economics, Nanning, 530003, P.R. China

[5] Guangxi Key Laboratory of Environmental Pollution Control Theory and Technology, Guilin University of Technology, Guilin, 541004, P.R. China

Summary:

Pages: 8; Figures: 2; Table: 1; Text: 2.

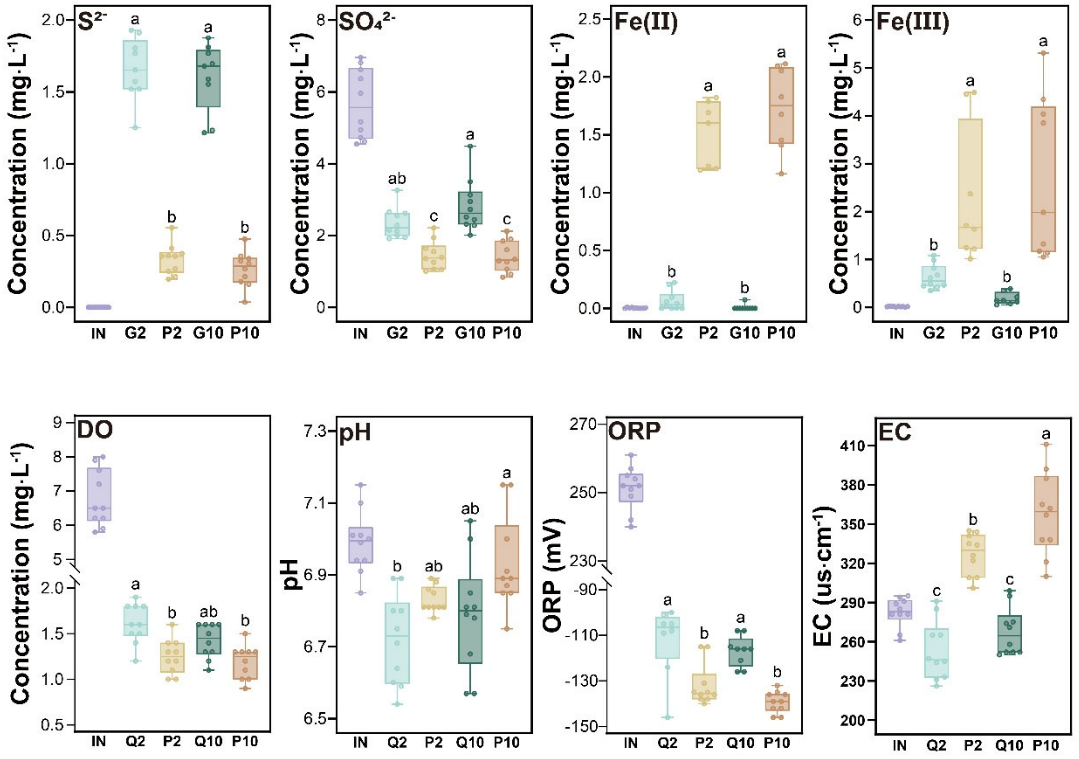

Figure S1. Concentrations of S^{2-}, SO_4^{2-}, Fe(II), Fe(III), and DO, pH, ORP, and EC in the CW microcosms during batch experiment (n = 10).

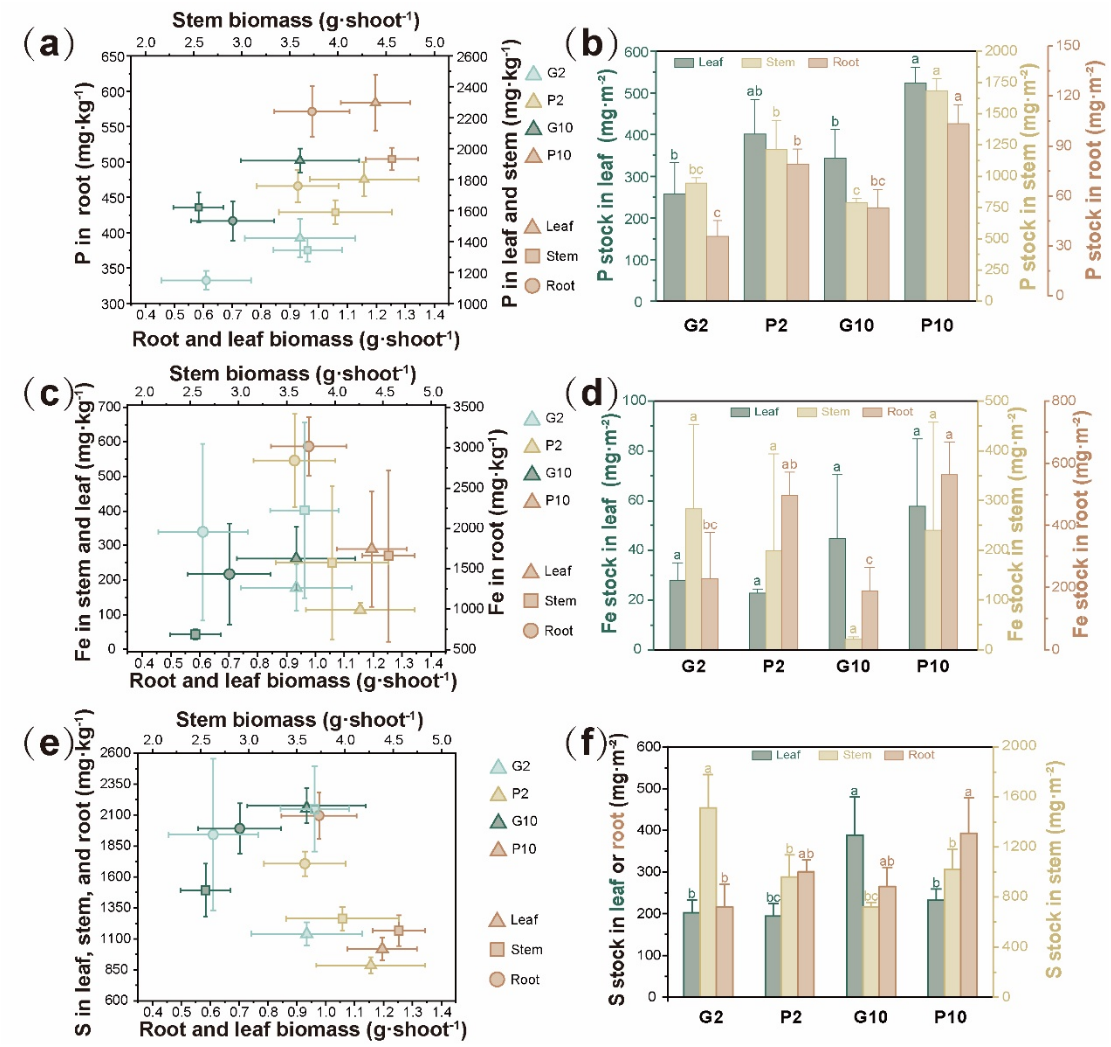

Figure S2. Biomass and phosphorus (a), iron (c), and sulfur (e) accumulation in root, stem, and leaves of *L. hexandra*. Phosphorus (b), iron (d), and sulfur (f) stock in root, stem, and leaves of *L. hexandra*.

Table S1. Bacterial community diversity indices in CWs.

Sample	Ace	Chao	Sobs	Shannon	Coverage
**G2**	826	820	813	4.25	0.9993
**P2**	1192	1185	1182	5.24	0.9993
**G10**	1202	1202	1202	5.30	1.0000
**P10**	1228	1223	1220	5.35	0.9994

Note: Ace, Chao, and Sobs indices were used to evaluate species richness, with higher values indicating greater community complexity. The Shannon index was used to assess species diversity, where higher values indicate greater diversity. Coverage reflects sequencing depth, and values above 0.99 generally indicate reliable data.

Text S1. Details of DNA extraction, Illumina MiSeq sequencing, and amplicon sequence processing.

DNA extraction and Illumina MiSeq sequencing

Total DNA was extracted from the microbial sample from each treatment using the E.Z.N.A.® soil DNA Kit (Omega Bio-tek, Norcross, GA, U.S.), according to the manufacturer's protocol. All DNA samples were quality checked and the concentration was quantified using a NanoDrop 2000 spectrophotometer (Thermo Fisher Scientific, Wilmington, DE, USA). Bacterial 16S rRNA gene fragments (V3-V4) were amplified from the extracted DNA using primers 515F (5′ - GTGYCAGCMGCCGCGGTAA - 3′) and 806R (5′ - GGACTACN-VGGGTWTCTAAT - 3′), and the following PCR conditions: 30 s at 95 °C, 30 s at 53 °C, and 45 s at 72 °C for 29 cycles. PCRs were performed with 4 μL 5 × TransStart FastPfu buffer, 2 μL 2.5 mM deoxynucleoside triphosphates (dNTPs), 0.8 μL of each primer (5 μM), 0.4 μL TransStart FastPfu DNA Polymerase, 10 ng of extracted DNA, and finally ddH2O to make up 20 μL. Agarose gel electrophoresis was performed to verify the size of amplicons. Amplicons were subjected to paired-end sequencing on the Illumina MiSeq sequencing platform using PE300 chemical at Majorbio Bio-Pharm Technology Co. Ltd. (Shanghai, China).

Amplicon sequence processing and analysis

After demultiplexing, the resulting sequences were merged with FLASH (v1.2.11)[1] and quality filtered with fastp (0.19.6) [2]. Then the high-quality

sequences were denoised using the DADA2 [3] plugin in the Qiime2 [4] (version 2020.2) pipeline with recommended parameters, which obtains single nucleotide resolution based on error profiles within samples. DADA2-denoised sequences are usually called amplicon sequence variants (ASVs). To minimize the effects of sequencing depth on alpha and beta diversity measurements, the number of sequences from each sample was rarefied to 4000, which still yielded an average Good's coverage of 97.90%. Taxonomic assignment of ASVs was performed using the Naive Bayes consensus taxonomy classifier implemented in Qiime2 and the SILVA 16S rRNA database (v138).

Text S2. The calculation of goodness-of-fit (GoF) in the PLS-SEM model.

According to Wetzels et al. [5], the GoF in the PLS-SEM model can be calculated as follows:

$$GoF = \sqrt{\overline{AVE} \times \overline{R^2}} = \sqrt{0.859 \times 0.761} = 0.809 \quad \text{(Eq. S1)}$$

where

$\overline{AVE}$ is the average variance extracted: square the factor loadings of all the reflective indicators, then calculate the average of these squared values;

$\overline{R^2}$ calculates the average of the R^2 values for all endogenous latent variables in the model.

References

1. Magoc, T. and Salzberg, S. L. FLASH: fast length adjustment of short reads to improve genome assemblies. Bioinformatics 2011, 27, 2957-2963. doi: 10.1093/bioinformatics/btr507
2. Chen, S.;Zhou, Y.;Chen, Y. and Gu, J. fastp: an ultra-fast all-in-one FASTQ preprocessor. Bioinformatics 2018, 34, 884-890. doi: 10.1093/bioinformatics/bty560
3. Callahan, B. J.;McMurdie, P. J.;Rosen, M. J.;Han, A. W.;Johnson, A. J. A. and Holmes, S. P. DADA2: High-resolution sample inference from Illumina amplicon data. Nature Methods 2016, 13, 581-583. doi: 10.1038/nmeth.3869
4. Bolyen, E.;Rideout, J. R.;Dillon, M. R.;Bokulich, N.;Abnet, C. C.;Al-Ghalith, G. A.;Alexander, H.;Alm, E. J.;Arumugam, M.;Asnicar, F.;Bai, Y.;Bisanz, J. E.;Bittinger, K.;Brejnrod, A.;Brislawn, C. J.;Brown, C. T.;Callahan, B. J.;Caraballo-Rodriguez, A. M.;Chase, J.;Cope, E. K.;Da Silva, R.;Diener, C.;Dorrestein, P. C.;Douglas, G. M.;Durall, D. M.;Duvallet, C.;Edwardson, C. F.;Ernst, M.;Estaki, M.;Fouquier, J.;Gauglitz, J. M.;Gibbons, S. M.;Gibson, D. L.;Gonzalez, A.;Gorlick, K.;Guo, J.;Hillmann, B.;Holmes, S.;Holste, H.;Huttenhower, C.;Huttley, G. A.;Janssen, S.;Jarmusch, A. K.;Jiang, L.;Kaehler, B. D.;Bin Kang, K.;Keefe, C. R.;Keim, P.;Kelley, S. T.;Knights, D.;Koester, I.;Kosciolek, T.;Kreps, J.;Langille, M. G. I.;Lee, J.;Ley, R.;Liu, Y.-X.;Loftfield, E.;Lozupone, C.;Maher, M.;Marotz, C.;Martin, B. D.;McDonald, D.;McIver, L. J.;Melnik, A. V.;Metcalf, J. L.;Morgan, S. C.;Morton, J. T.;Naimey, A. T.;Navas-Molina, J. A.;Nothias, L. F.;Orchanian, S. B.;Pearson, T.;Peoples, S. L.;Petras, D.;Preuss, M. L.;Pruesse, E.;Rasmussen, L. B.;Rivers, A.;Robeson, M. S., II;Rosenthal, P.;Segata, N.;Shaffer, M.;Shiffer, A.;Sinha, R.;Song, S. J.;Spear, J. R.;Swafford, A. D.;Thompson, L. R.;Torres, P. J.;Trinh, P.;Tripathi, A.;Turnbaugh, P. J.;Ul-Hasan, S.;vander Hooft, J. J. J.;Vargas, F.;Vazquez-Baeza, Y.;Vogtmann, E.;von Hippel, M.;Walters, W.;Wan, Y.;Wang, M.;Warren, J.;Weber, K. C.;Williamson, C. H. D.;Willis, A. D.;Xu, Z. Z.;Zaneveld, J. R.;Zhang, Y.;Zhu, Q.;Knight, R. and Caporaso, J. G. Reproducible, interactive, scalable and extensible microbiome data science using QIIME 2. Nature Biotechnology 2019, 37, 852-857. doi: 10.1038/s41587-019-0209-9
5. Wetzels, M.;Odekerken-Schroder, G. and van Oppen, C. Using PLS Path Modeling for Assessing Hierarchical Construct Models: Guidelines and Empirical Illustration. MIS Q. 2009, 33, 177-195. doi: 10.2307/20650284
